# Musical Instrument Identification Using Deep Learning Approach

**DOI:** 10.3390/s22083033

**Published:** 2022-04-15

**Authors:** Maciej Blaszke, Bożena Kostek

**Affiliations:** 1 Multimedia Systems Department, Faculty of Electronics, Telecommunications and Informatics, Gdańsk University of Technology, Narutowicza 11/12, 80-233 Gdańsk, Poland; mblaszke@multimed.org; 2 Audio Acoustics Laboratory, Faculty of Electronics, Telecommunications and Informatics, Gdańsk University of Technology, Narutowicza 11/12, 80-233 Gdańsk, Poland

**Keywords:** deep learning, musical instrument identification, musical information retrieval

## Abstract

The work aims to propose a novel approach for automatically identifying all instruments present in an audio excerpt using sets of individual convolutional neural networks (CNNs) per tested instrument. The paper starts with a review of tasks related to musical instrument identification. It focuses on tasks performed, input type, algorithms employed, and metrics used. The paper starts with the background presentation, i.e., metadata description and a review of related works. This is followed by showing the dataset prepared for the experiment and its division into subsets: training, validation, and evaluation. Then, the analyzed architecture of the neural network model is presented. Based on the described model, training is performed, and several quality metrics are determined for the training and validation sets. The results of the evaluation of the trained network on a separate set are shown. Detailed values for precision, recall, and the number of true and false positive and negative detections are presented. The model efficiency is high, with the metric values ranging from 0.86 for the guitar to 0.99 for drums. Finally, a discussion and a summary of the results obtained follows.

## 1. Introduction

The identification of complex audio, including music, has proven to be complicated. This is due to the high entropy of the information contained in audio signals, wide range of sources, mixing processes, and the difficulty of analytical description, hence the variety of algorithms for the separation and identification of sounds from musical material. They mainly use spectral and cepstral analyses, enabling them to detect the fundamental frequency and their harmonics and assign the retrieved patterns to a particular instrument. However, this comes with some limitations, at the expense of increasing temporal resolution, frequency resolution decreases, and vice versa. In addition, it should be noted that these algorithms do not always allow the extraction of percussive tones and other non-harmonic effects, which may therefore constitute a source of interference for the algorithm, which may hinder its operation and reduce the accuracy and reliability of the result.

Moreover, articulation such as glissando or tremolo causes frequency shifts in the spectrum; transients may generate additional components in the signal spectrum. Another important factor should be kept in mind: music in Western culture is based—to some extent—on consonances, which, although pleasing to the ear, are based on frequency ratios to fundamental tones. Thus, an obvious consequence is the overlap of harmonic tones in the spectrum, which creates a problem for most algorithms.

It should be remembered that recording musical instruments requires sensors. It is of enormous importance how a particular instrument is recorded. Indeed, the acoustic properties of musical instruments, researched theoretically for many epochs, as well as sound engineering practice, prescribe how to register an instrument in a given environment and conditions almost perfectly. These were the days of music recording in studios with acoustics designed for that purpose or registering music during a live concert with a lot of expertise on what microphones to use. On that basis, identifying a musical instrument sound within a recording is reasonably affordable both in terms of a human ear and automatic recognition. However, music instrument recording and its processing have changed over the last few decades. Nowadays, music is recorded everywhere and with whatever sensors are available, including smartphones. As a consequence, the task of the automated identification process became both much more intensive and necessary. This is because identifying musical instruments is of importance in many areas no longer closely related to music, i.e., automatically creating sound for games, organizing music social services, separating music mixes into tracks, amateur recordings, etc. Moreover, instruments may become sensors addressing an interesting concept: could the sound of a musical instrument be used to infer information about the instrument’s physical properties [1]? This is based on the notion that any vibrating instrument body part may be used for measuring its physical properties. Building new interfaces for musical expression (NIME) is another paradigm related to new sonic creation and a new way of musical instrument sound expression and performance [2]. Last but not least, smart musical instruments, a class of IoT (Internet of Things) devices, should be mentioned in the context of music creation [3]. Turchet et al., devised a sound engine incorporating digital signal processing, sensor fusion, and embedded machine learning techniques to classify the position, dynamics, and timbre of each hit of a smart cajón [4].

Overall, both classical and sensor-based instruments need to be subject to sound identification and further applications, e.g., computational auditory scene analysis (CASA), human–computer interaction (HCI), music post-production, music information retrieval, automatic music mixing, music recommendation systems, etc. The identification of various instruments in the music mix, as well as the retrieval of melodic lines, belongs to the task of automatic music transcription (AMT) systems [5]. This also concerns blind source separation (BSS) [6,7]. Moreover, some other methods should be cited as they constitute the basis of BSS, e.g., independent component analysis (ICA) [8] or empirical mode decomposition (EMD) [9].

However, the problem in some of the analyzed cases is the classification: assigning the analyzed sample to a specific class, that is, in this case, the musical instrument. The work aims to propose an algorithm for automatic identification of all instruments present in an audio excerpt using sets of individual convolutional neural networks (CNN) per tested instrument. The motivation for this work was the need for a flexible model where any instrument could be added to the previously trained neural network. The novelty of the proposed solution lies in splitting the model into separate processing paths, one per instrument to be identified. Such a solution allows using models with various architecture complexity for different instruments, adding new submodels to the previously trained model, or replacing one instrument for another.

The paper starts with a review of tasks related to musical instrument identification. It focuses on the tasks performed, input type, algorithms employed, and metrics used. The main part of the study shows the dataset prepared for the experiment and its division into subsets: training, validation, and evaluation. The following section presents the analyzed architecture of the neural network model and its flexibility to expand. Based on the described model, training is performed, and several identification quality metrics are determined for training and validation sets. Then, the results of the evaluation of the trained network on a separate set are shown. Finally, a discussion and a summary of the results obtained follows.

## 2. Study Background

### 2.1. Metadata

The definition of metadata refers to data that provides information about other data. Metadata is also one of the basic sources of information about songs and audio samples. The ID3v2 informal standard [10] evolved from the ID3 tagging system, and it is a container of additional data embedded in the audio stream. Besides the typical parameters of the signal based on the MPEG-7 standard [11], information such as the performer, music genre, the instruments used, etc., usually appears in the metadata [12,13].

While in the case of newly created songs, individual sound examples, and music datasets, this information is already inserted in the audio file, older databases may not have such metadata tags. This is of particular importance when the task considered is to name all musical instruments present in a song by retrieving an individual stem from an audio file [14,15,16,17]. To this end, two approaches are still seen in this research area. The first consists of extracting a feature vector (FV) containing audio descriptors and using the baseline machine learning algorithms [12,15,16,17,18,19,20,21,22,23,24,25,26,27]. The second is based on the 2D audio representation and a deep learning model [28,29,30,31,32,33,34,35,36,37,38,39,40,41], or a more automated version when a variational or deep softmax autoencoder is used for the audio representation retrieval [32,42]. Therefore, by employing machine learning, it is possible to implement a classifier for particular genres or instrument recognition.

An example of a precisely specified feature vector in the audio domain is the MEPG-7 standard, described in ISO/IEC 15938 [11]. It contains descriptors divided into six main groups:

1. Basic: based on the value of the audio signal samples;
2. BasicSpectral: simple time–frequency signal analysis;
3. SpectralBasis: one-dimensional spectral projection of a signal prepared primarily to facilitate signal classification;
4. SignalParameters: information about the periodicity of the signal;
5. TimbralTemporal: time and musical timbre features;
6. TimbralSpectral: description of the linear–frequency relationships in the signal.

Reviewing the literature that describes the classification of musical instruments, it can be seen that this has been in development for almost three decades [17,18,25,28,36,41]. These works use various sets of signals and statistical parameters for the analyzed samples, standard MPEG-7 descriptors, spectrograms, mel-frequency cepstral coefficients (MFCC), or constant-Q transform (CQT)—the basis for their operation. Similar to the input data, the baseline algorithms employed for classification also differ. They are as follows: HMM (hidden Markov model), k-NN (k-nearest neighbors) classifier, SOM (self-organizing map), SVM (support vector machine), decision trees, etc. Depending on the FVs and algorithms applied, they achieve an efficiency of even 99% for musical instrument recognition. However, as already said, some issues remain, such as instruments with differentiated articulation. The newer studies refer to deep models; however, the outcome of these works varies between works.

### 2.2. Related Work

Musical instrument identification also has a vital role in various classification tasks in audio fields. One such example is genre classification. In this context, many algorithms were used but obtained similar results. It should be noted that a music genre is conditioned by the instruments present in a musical piece. For example, the cello and saxophone are often encountered in jazz music, whereas the banjo is almost exclusively associated with country music. In music genre classification, several well-known techniques have been used, such as SVM (support vector machine) [14,19,25,26,33], ANN (artificial neural networks) [24,40], etc., as well as CNN (convolutional neural networks) [28,30,34,35,36,37,38,39,40], RNN (recurrent neural networks) [28,41], and CRNN (convolutional recurrent neural network) [31,34].

Table 1 shows an overview of various algorithms and tasks described above along with the obtained results [14,15,16,18,19,20,21,22,23,24,25,26,27,28,29,30,31,33,34,35,36,37,38,39,40,41].

As already mentioned, the aim of this study is to build an algorithm for automatic identification of instruments present in an audio excerpt using sets of individual convolutional neural networks (CNN) per tested instrument. Therefore, a flexible model where any instrument could be added to the previously trained neural network should be created.

## 3. Dataset

**Figure 1:**
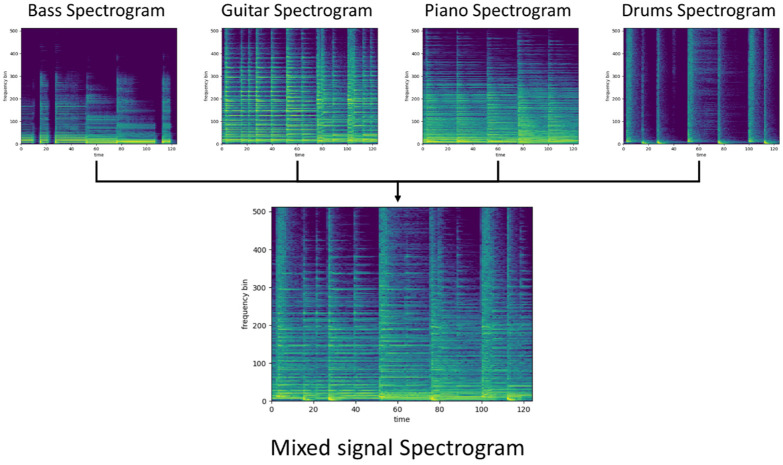
Example of spectrograms of selected instruments and the prepared mix.

In our study, the Slakh dataset was used, which contains 2100 audio tracks with aligned MIDI files, and separate instrument stems along with tagging [43]. From all of the available instruments, four were selected for the experiment: bass, drums, guitar, and piano. After selection, each song was split into 4-second excerpts. If the level of instrument signal in the extracted part was lower than −60 dB, then this instrument was excluded from the example. This made it possible to decrease computing costs and increase the instrument count in the mix variability. Additionally, each part has a randomly selected gain for all instruments separately. An example of spectrograms of selected instruments and the prepared mix are presented in Figure 1.

The examples were then stored using the NumPy format on files that contain mixed signals, instrument references, and vectors of labels to indicate which instruments were used in the mix [44].

To achieve repeatability of the training results, the whole dataset was a priori divided into three parts, but with the condition that a single audio track cannot be split into each part:

1. Training set—116,413 examples;
2. Validation set—5970 examples;
3. Evaluation set—6983 examples.

The number of individual instrument appearances in the mix is not similar, to not favor any of them. A class weighting vector is passed to the training algorithm to balance the results between instruments. Calculated weights are as follows:

4. Bass—0.65
5. Guitar—1.0
6. Piano—0.78
7. Drums—0.56

**Figure 2:**
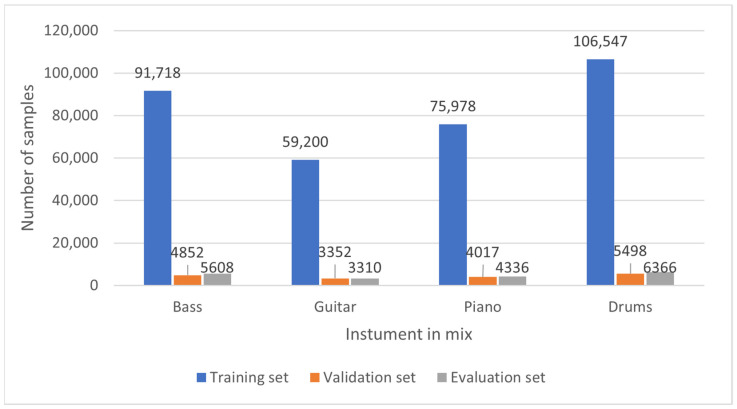
Histogram of the instrument classes in the mixes.

**Figure 3:**
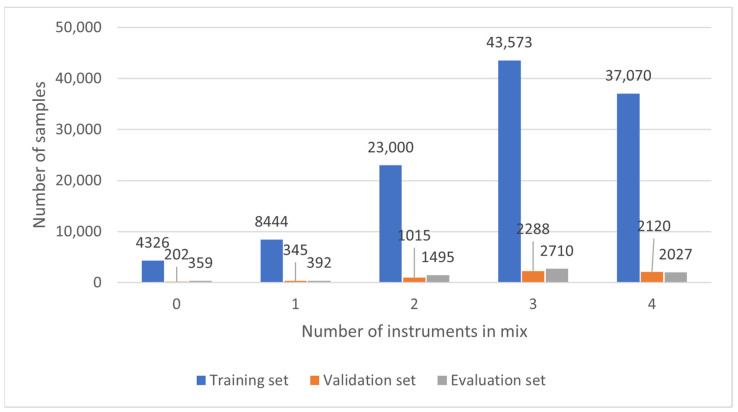
Histogram of instruments in the mixes.

Furthermore, the number of instruments in a given sample also varies. Due to the structure of music pieces, the largest part (about 1/3 of all of the examples in the dataset) contains three instruments. Four, two, and then one instrument populate the remaining parts. In addition, music samples that do not have any instrument are introduced to the algorithm input to train the system to understand that such a case can also occur. Histograms of the instrument classes in the mixes and the number of instruments in a mix are presented in Figure 2 and Figure 3.

## 4. Model

The proposed neural network was implemented using the Keras framework and functional API [45]. The model initially produces MFCC (mel frequency cepstral coefficients) from the raw audio signal using built-in Keras methods [46]. The parameters for those operations are as follows:

- 1024 samples Hamming window length;
- 512 samples window step;
- 40 MFCC bins.

**Figure 4:**
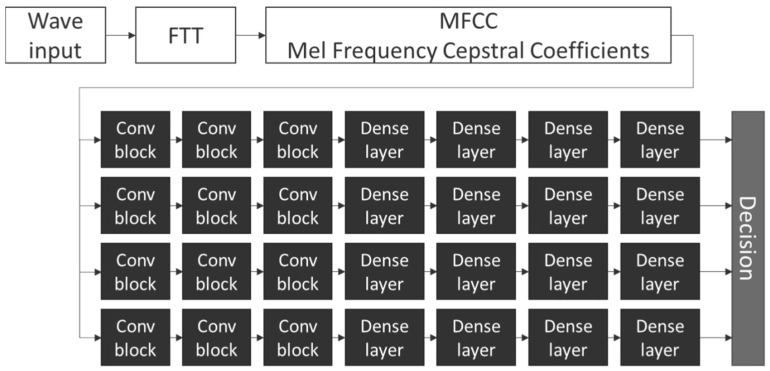
Model architecture.

In contrast to other methods where a single model performs identification or classification of all instruments, the used model employs sets of individual identically defined submodels—one per instrument. The proposed architecture contains 2-dimensional convolution layers in the beginning. The number of filters was, respectively, 128, 64, and 32 with (3, 3) kernels and the ReLU activation function [47]. In addition, 2-dimensional max pulling and batch normalization are incorporated into the model after each convolution [48,49,50]. To obtain the decision, four dense layers were used with 64, 32, 16, and 1 unit, respectively [50]. The model contains 706,182 trainable parameters. The topology of the network is presented in Figure 4.

The simplified code for model preparation is presented below. Each instrument has its own model preparation function, where a new model could be created, or a pre-trained model could be loaded. In the last operation, outputs from all models are concatenated and set as a whole model output.

def prepareModel(input_shape):

           dense_outputs = []
           input = Input(shape = input_shape)
           mfcc = prepareMfccModel(input)
           dense_outputs.append(prepareBassModel(mfcc))
           dense_outputs.append(prepareGuitarModel(mfcc))
           dense_outputs.append(preparePianoModel(mfcc))
           dense_outputs.append(prepareDrumsModel(mfcc))
           concat = Concatenate()(dense_outputs)
           model = Model(inputs = input, outputs = concat)
           return model

### 4.1. Training

The training was performed using the Tensorflow framework for the Python language. The model was trained for 100 epochs with the mean squared error (MSE) as the loss function. Additionally, during training, the precision, recall, and AUC ROC (area under the receiver operating characteristic curve) were calculated. The best model was selected based on the AUC ROC metric [51]. Precision is a ratio of true positive examples to all examples identified as an examined class. The definition of this metric is presented in Equation (1) [52].

(1) $$precision=\frac{True\;Positives}{True\;Positives+False\;Positives}$$

The recall ratio of true positive examples to all examples in the examined class is defined by Equation (2):

(2) $$recall=\frac{True\;Positives}{True\;Positives+False\;Negatives}$$

Additionally, for evaluation purposes, the F1 score was used [53]. This metric represents a harmonic mean of precision and recall. The exact definition is presented in Equation (3) [52].

(3) $$F1\;score=2\cdot \frac{precision\cdot recall}{precision+recall}$$

**Figure 5:**
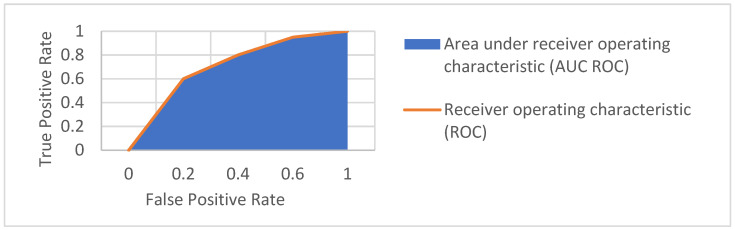
Example of the receiver operating characteristic and area under the curve.

The receiver operating characteristic (ROC) shows a trade-off between true and false positive results in the function of various decision thresholds. The ROC and AUC ROC are illustrated in Figure 5. We included this illustration to visualize the importance of true and false positives in the identification process. 

**Figure 6:**
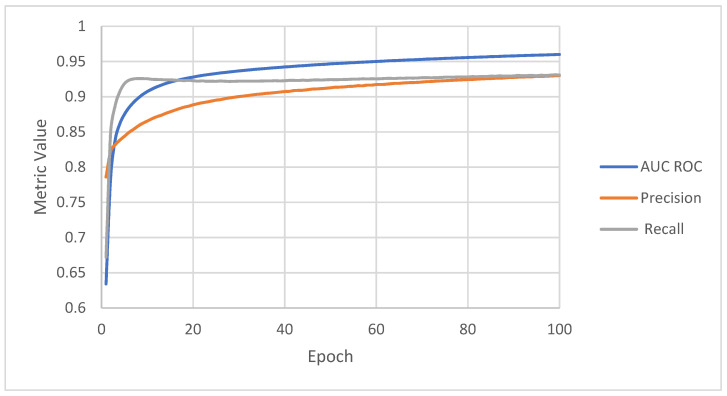
Metrics achieved by the algorithm on the training set.

**Figure 7:**
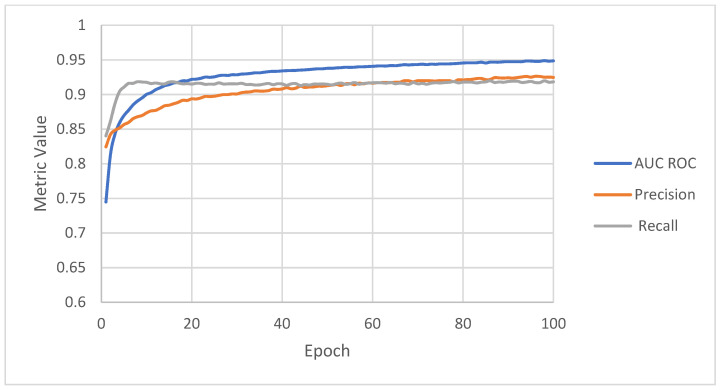
Metrics achieved by the algorithm on the validation set.

Precision, recall, and AUC ROC calculated during training are presented in Figure 6 and Figure 7. On the training set, recall starts from 0.67 and increases to 0.93. Precision starts from a higher value, 0.78, and increases throughout the whole training process to 0.93. AUC ROC builds up from the lowest value, 0.63, but increases to the highest value, 0.96. The values of the metrics for the validation sets look similar to those of the training set. During training, both metrics increase to 0.95 and 0.97 for the training set and, respectively, 0.95 and 0.94 for the validation set. The recall starts from 0.84 and increases to 0.93, precision from 0.82 to 0.92, and AUC ROC from 0.74 to 0.95.

The training was performed using a single RTX2070 graphics card with an AMD Ryzen 5 3600 processor and 32 GB of RAM. The duration of a single epoch is about 8 min using multiprocessing data loading and with a batch size of 200.

### 4.2. Evaluation Results and Discussion

The evaluation was carried out using a set of 6983 examples prepared from audio tracks not presented in the training and validation sets. The processing time for a single example was about 0.44 s, so the algorithm works approx. 10 times faster than real-time. The averaged results for individual metrics are as follows:

- Precision—0.92;
- Recall—0.93;
- AUC ROC—0.96;
- F1 score—0.93.

The individual components of precision and recall are as follows:

- True positive—17,759;
- True negative—6610;
- False positive—1512;
- False negative—1319.

**Figure 8:**
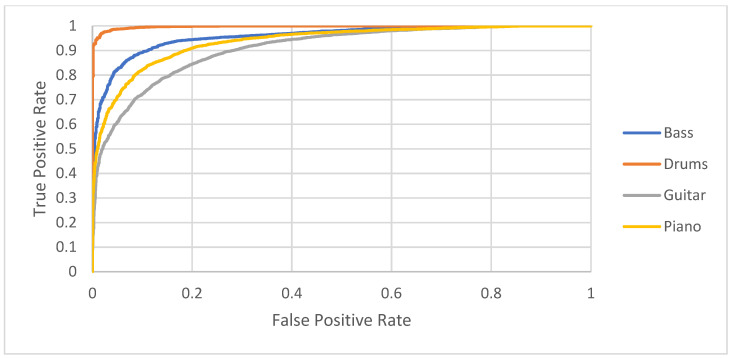
ROC curves for each instrument tested.

Based on the results obtained, more detailed analyses were also carried out, discerning individual instruments. The ROC curves are presented in Figure 8. They indicate that the most easily identifiable class is percussion, which can obtain a true positive rate of 0.95 for a relatively low false positive rate of about 0.01. The algorithm is slightly worse at identifying bass because to achieve similar effectiveness, the false positive rate for the bass would have to be 0.2. When it comes to guitar and piano, to achieve effectiveness of about 0.9, one has to accept a false positive rate of 0.27 and 0.19, respectively.

Detailed values for precision, recall, and the number of true and false positive and negative detections are presented in Table 2. By comparing these results with the ROC plot in Figure 8, one can see confirmation that the model is more capable of recognizing drums and also bass. Looking at the metric values for guitar, one can see that the model has a similar trend when resulting in the samples received as false negatives and false positives. For the piano, the opposite happens, i.e., more samples are marked as false positives. Table 3 presents the confusion matrix.

### 4.3. Redefining the Models

Using the ability of the model’s infrastructure to easily swap entire blocks for individual instruments, an additional experiment was conducted. Submodels for drums and guitar were changed to smaller and bigger ones. A detailed comparison of the block structure before and after the changes introduced is shown in Table 4.

**Figure 9:**
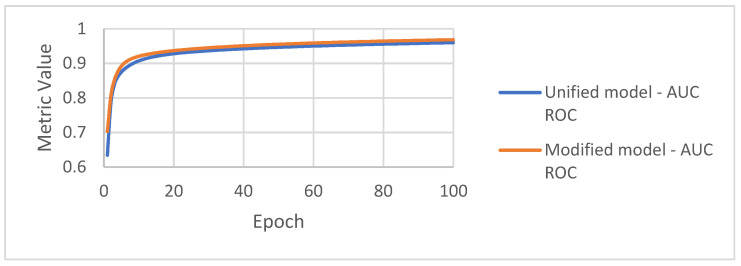
AUC ROC achieved by the algorithms on the training set.

**Figure 10:**
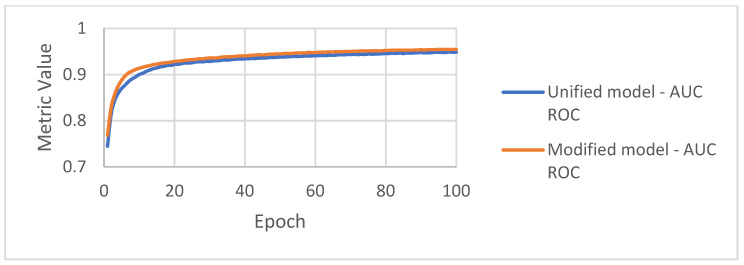
AUC ROC achieved by the algorithms on the validation set.

AUC ROC curves calculated during the training and validation stages are presented in Figure 9 and Figure 10. The training and validation curves look similar, but the modified model achieves better results by about 0.01.

### 4.4. Evaluation Result Comparison

The evaluation of the new model was prepared based on the same conditions as in Section 4.2. A comparison of results for the first submodel and models after modifications is presented in Table 5, whereas Table 6 shows results per modified instrument. Because of rounding metric values to two decimal places, the differences are not strongly visible when looking at the entire evaluation set. However, comparing true positive, true negative, and false positive, all those measures are higher on the modified model than on the unified model, namely, of about 200 examples. The only value of the false negative examples is worse in 61 examples. Looking at the results of changed instruments, one can see that the smaller model for drums performs similarly compared to the unified model. A larger model obtained for guitar presents better results on precision and F1 score.

**Figure 11:**
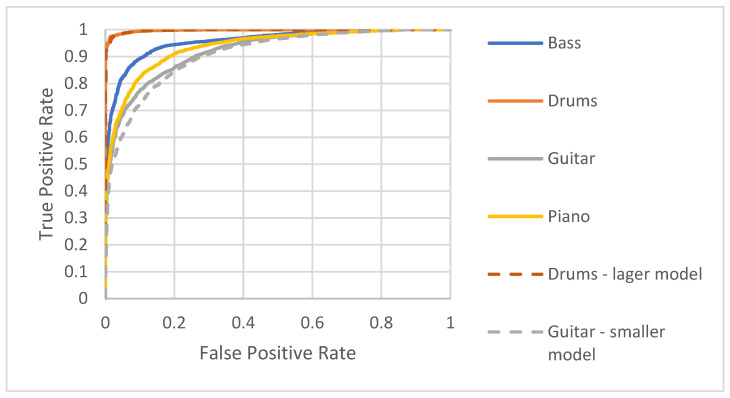
ROC curves for each instrument tested on the unified and modified models.

The ROC curves for the unified and modified models are presented in Figure 11. Focusing on the modified models for drums and guitar, it could be noticed that the smaller model for drums has an almost identical shape to the ROC curve. In contrast, the guitar model shows better results using a bigger model, e.g., the true positive rate increases from 0.72 to 0.77, whereas false positive rate equals 0.1.

**Figure 12:**
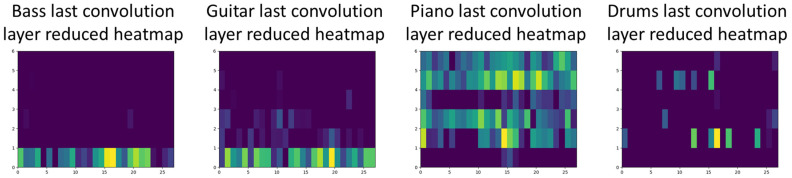
Reduced heatmaps for the last convolutional layers per identified instrument.

Figure 12 presents reduced heatmaps for the last convolutional layers per identified instrument for one of the examples from the evaluation dataset. Comparing heatmaps between each other, one can see that the bass model focuses mainly on lower frequencies for the whole signal, guitar on low and mid frequencies, piano on mid and high frequencies but also the whole signal, and finally, drums for all of the frequencies and short-time signals.

## 5. Discussion

The presented results show that it is possible to determine the instruments present in a given excerpt of a musical recording with a precision of 93% and an F1 score of 0.93 using a simple convolutional network based on the MFCC.

The experiment also shows that the effectiveness of identification depends on the instrument tested. The drums are more easily identifiable, while the guitar and piano produced worse results.

The current state of the art in audio recognition fields focuses on single or predominant instrument recognition and genre classification. With regard to the results of those tasks, an accuracy of about 100% can be found, but when looking at musical instrument recognition results, the metric values are lower, e.g., AUC ROC of approximately 0.91 [37] or F1 score of about 0.64 [36]. The proposed solution can achieve an AUC ROC of about 0.96 and an F1 score of about 0.93, outperforming the other methods.

An additional difference compared to state-of-the-art methods is the flexibility of the model. The presented results show that an operation of a submodel switch allows, for example, reducing the size of the model in the case when the instrument is readily identifiable without affecting the architecture of the other identifiers. Thus, it is possible to save computational power compared to a model with a large, unified architecture. On the other hand, the submodel can be increased to improve the results for an instrument presenting poorer quality without affecting the other instruments under the study.

## 6. Conclusions

The novelty of the proposed solution lies in the model architecture, where every instrument has an individual and independent identification path. It produces outputs focused on specific patterns in the MFCC signal depending on the examined instrument, opposite to state-of-the-art methods, where a single convolutional part obtains one pattern per all instruments.

The proposed framework is very flexible, so it could use instrument models with various complexity—more advanced for those with weaker results and more straightforward for those with better results. Another advantage of this flexibility is the opportunity to extend the model with more instruments by adding new submodels in the architecture proposed. This thread will be pursued further, especially as a new dataset is being prepared that will contain musical instruments that are underrepresented in music repositories, i.e., the harp, Rav vast, and Persian cymbal (santoor). Recordings of these instruments are created with both dynamic and condenser microphones at various distances and angles of microphone positioning, and they will be employed for creating new submodels in the identification system.

Additionally, the created model will be worked on toward on-the-fly musical instrument identification as this will enable its broader applicability in real-time systems.

Moreover, we may use other neural network structures as known in the literature [54,55], e.g., using sample-level filters instead of frame-level input representations [56], and trying other approaches to music feature extraction, e.g., including derivation of rhythm, melody, and harmony and determining their weights by employing the exponential analytic hierarchy process (AHP) [57]. Lastly, the model proposed may be tested with audio signals other than music, such as classification of urban sounds [58].

## Figures and Tables

**Table 1:** Related work.

Authors	Year	Task	Input Type	Algorithm	Metrics
Avramidis K., Kratimenos A., Garoufis C., Zlatintsi A., Maragos P. [28]	2021	Predominant instrument recognition	Raw audio	RNN (recurrent neural networks), CNN (convolutional neural networks), and CRNN (convolutional recurrent neural network)	LRAP (label ranking average precision)—0.747 F1 micro—0.608 F1 macro—0.543
Kratimenos A., Avramidis K., Garoufis C., Zlatintsi, A., Maragos P. [36]	2021	Instrument identification	CQT (constant-Q transform)	CNN	LRAP—0.805 F1 micro—0.647 F1 macro—0.546
Zhang F. [41]	2021	Genre detection	MIDI music	RNN	Accuracy—89.91% F1 macro—0.9
Shreevathsa P. K., Harshith M., A. R. M. and Ashwini [40]	2020	Single instrument classification	MFCC (mel-frequency cepstral coefficient)	ANN (artificial neural networks) and CNN	ANN accuracy—72.08% CNN accuracy—92.24%
Blaszke M., Koszewski D., Zaporowski S. [30]	2019	Single instrument classification	MFCC	CNN	Precision—0.99 Recall—1.0 F1 score—0.99
Das O. [33]	2019	Single instrument classification	MFCC and WLPC (warped linear predictive coding)	Logistic regression and SVM (support vector machine)	Accuracy—100%
Gururani S., Summers C., Lerch A. [34]	2018	Instrument identification	MFCC	CNN and CRNN	AUC ROC—0.81
Rosner A., Kostek B. [26]	2018	Genre detection	FV (feature vector)	SVM	Accuracy—72%
Choi K., Fazekas G., Sandler M., Cho K. [31]	2017	Audio tagging	MFCC	CRNN (convolutional recurrent neural network)	ROC AUC (receiver operator characteristic)—0.65-0.98
Han Y., Kim J., Lee K. [35]	2017	Predominant instrument recognition	MFCC	CNN	F1 score macro—0.503 F1 score micro—0.602
Pons J., Slizovskaia O., Gong R., Gómez E., Serra X. [39]	2017	Predominant instrument recognition	MFCC	CNN	F1 score micro—0.503 F1 score macro—0.432
Bhojane S.B., Labhshetwar O.G., Anand K., Gulhane S.R. [29]	2017	Single instrument classification	FV (MIR Toolbox)	k-NN (k-nearest neighbors)	A system that can listen to the musical instrument tone and recognize it (no metrics shown)
Lee J., Kim T., Park J., Nam J. [37]	2017	Instrument identification	Raw audio	CNN	AUC ROC—0.91 Accuracy—86% F1 score—0.45%
Li P., Qian J., Wang T. [38]	2015	Instrument identification	Raw audio, MFCC, and CQT (constant-Q transform)	CNN	Accuracy—82.74%
Giannoulis D., Benetos E., Klapuri A., Plumbley M. D. [20]	2014	Instrument identification	CQT (constant-Q transform of a time domain signal)	Missing feature approach with AMT (automatic music transcription)	F1—0.52
Giannoulis D., Klapuri A., [21]	2013	Instrument recognition in polyphonic audio	A variety of acoustic features	Local spectral features and missing-feature techniques, mask probability estimation	Accuracy—67.54%
Bosch J. J., Janer J., Fuhrmann F., Herrera P. [14]	2012	Predominant instrument recognition	Raw audio	SVM	F1 score micro—0.503 F1 score macro—0.432
Heittola T., Klapuri A., Virtanen T. [16]	2009	Instrument recognition in polyphonic audio	MFCC	NMF (non-negative matrix factorization) and GMM	F1 score—0.62
Essid S., Richard G., David B. [19]	2006	Single instrument classification	MFCC and FV	GMM (Gaussian mixture model) and SVM	Accuracy—93%
Kostek B. [23]	2004	Single instrument classification (12 instruments)	Combined MPEG-7 and Wavelet-Based FVs	ANN	Accuracy—72.24%
Eronen A. [15]	2003	Single instrument classification	MFCC	ICA (independent component analysis) ML and HMM (hidden Markov model)	Accuracy between: 62–85%
Kitahara T., Goto M., Okuno H. [22]	2003	Single instrument classification	FV	Discriminant function based on the Bayes decision rule	Recognition rate—79.73%
Tzanetakis G., Cook P. [27]	2002	Genre detection	FV and MFCC	SPR (subtree pruning–regrafting)	Accuracy—61%
Kostek B., Czyżewski A. [24]	2001	Single instrument classification	FV	ANN	Accuracy—94.5%
Eronen A., Klapuri A. [18]	2000	Single instrument classification	FV	k-NN	Accuracy—80%
Marques J., Moreno P. J. [25]	1999	Single instrument classification	MFCC	GMM and SVM	Error rate—17%

**Table 2:** Results per instrument.

Metric	Bass	Drums	Guitar	Piano
Precision	0.94	0.99	0.82	0.87
Recall	0.94	0.99	0.82	0.91
F1 score	0.95	0.99	0.82	0.89
True positive	5139	6126	2683	3811
True negative	1072	578	2921	2039
False positive	288	38	597	589
False negative	301	58	599	361

**Table 3:** Confusion matrix (in percentage points).

		Ground Truth Instrument [%]			
		Bass	Guitar	Piano	Drums
Predicted instrument	Bass	81	8	7	0
	Guitar	4	69	13	0
	Piano	5	12	77	0
	Drums	3	7	6	82

**Table 4:** Comparison between the first submodel and models after modification.

Block Number	Unified Submodel	Guitar Submodel	Drums Submodel
1	2D convolution: Kernel—3 × 3 Filters—128 2D Max pooling: Kernel 2 × 2 Batch Normalization	2D convolution: Kernel—3 × 3 Filters—256 2D Max pooling: Kernel 2 × 2 Batch Normalization	2D convolution: Kernel—3 × 3 Filters—64 2D Max pooling: Kernel 2 × 2 Batch Normalization
2	2D convolution: Kernel—3 × 3 Filters—62 2D Max pooling: Kernel 2 × 2 Batch Normalization	2D convolution: Kernel—3 × 3 Filters—128 2D Max pooling: Kernel 2 × 2 Batch Normalization	2D convolution: Kernel—3 × 3 Filters—32 2D Max pooling: Kernel 2 × 2 Batch Normalization
3	2D convolution: Kernel—3 × 3 Filters—32 2D Max pooling: Kernel 2 × 2 Batch Normalization	2D convolution: Kernel—3 × 3 Filters—64 2D Max pooling: Kernel 2 × 2 Batch Normalization	2D convolution: Kernel—3 × 3 Filters—16 2D Max pooling: Kernel 2 × 2 Batch Normalization
4	Dense Layer: Units—64	Dense Layer: Units—64	Dense Layer: Units—64
5	Dense Layer: Units—32	Dense Layer: Units—32	Dense Layer: Units—32
6	Dense Layer: Units—16	Dense Layer: Units—16	Dense Layer: Units—16
7	Dense Layer: Units—1	Dense Layer: Units—1	Dense Layer: Units—1

**Table 5:** Comparison between the first submodel and models after modifications.

Metric	Unified Model	Modified Model
Precision	0.92	0.93
Recall	0.93	0.93
AUC ROC	0.96	0.96
F1 score	0.93	0.93
True positive	17,759	17,989
True negative	6610	6851
False positive	1512	1380
False negative	1319	1380

**Table 6:** Results per modified instrument models.

	Drums		Guitar	
Metric	Unified Model	Modified Model	Unified Model	Modified Model
Precision	0.99	0.99	0.82	0.86
Recall	0.99	0.99	0.82	0.8
F1 score	0.99	0.99	0.82	0.83
True positive	6126	6232	2683	2647
True negative	578	570	2921	3150
False positive	38	47	597	444
False negative	58	51	599	659

## Data Availability

Not applicable.
